# Mastering the Canonical Loop of Serine Protease Inhibitors: Enhancing Potency by Optimising the Internal Hydrogen Bond Network

**DOI:** 10.1371/journal.pone.0019302

**Published:** 2011-04-27

**Authors:** Joakim E. Swedberg, Simon J. de Veer, Kei C. Sit, Cyril F. Reboul, Ashley M. Buckle, Jonathan M. Harris

**Affiliations:** 1 Institute of Health and Biomedical Innovation, Queensland University of Technology, Brisbane, Queensland, Australia; 2 Department of Biochemistry and Molecular Biology, School of Biomedical Sciences, Faculty of Medicine and Victorian Bioinformatics Consortium, Monash University, Clayton, Victoria, Australia; University of Queensland, Australia

## Abstract

**Background:**

Canonical serine protease inhibitors commonly bind to their targets through a rigid loop stabilised by an internal hydrogen bond network and disulfide bond(s). The smallest of these is sunflower trypsin inhibitor (SFTI-1), a potent and broad-range protease inhibitor. Recently, we re-engineered the contact β-sheet of SFTI-1 to produce a selective inhibitor of kallikrein-related peptidase 4 (KLK4), a protease associated with prostate cancer progression. However, modifications in the binding loop to achieve specificity may compromise structural rigidity and prevent re-engineered inhibitors from reaching optimal binding affinity.

**Methodology/Principal Findings:**

In this study, the effect of amino acid substitutions on the internal hydrogen bonding network of SFTI were investigated using an *in silico* screen of inhibitor variants in complex with KLK4 or trypsin. Substitutions favouring internal hydrogen bond formation directly correlated with increased potency of inhibition *in vitro*. This produced a second generation inhibitor (SFTI-FCQR Asn_14_) which displayed both a 125-fold increased capacity to inhibit KLK4 (*K*_i_ = 0.0386±0.0060 nM) and enhanced selectivity over off-target serine proteases. Further, SFTI-FCQR Asn_14_ was stable in cell culture and bioavailable in mice when administered by intraperitoneal perfusion.

**Conclusion/Significance:**

These findings highlight the importance of conserving structural rigidity of the binding loop in addition to optimising protease/inhibitor contacts when re-engineering canonical serine protease inhibitors.

## Introduction

Prostate cancer is the most commonly diagnosed male cancer in western countries, accounting for more than 32,000 deaths last year in the United States alone [1]. Although current treatments for localized prostate cancer are highly successful, less than one third of patients with metastatic disease survive five years following diagnosis [1]. This emphasises the urgent need for effective treatments for patients suffering from late stage disease. Prostate cancer is primarily detected using serum levels of kallikrein-related peptidase 3 (KLK3, prostate-specific antigen, PSA), which is the established biomarker for diagnosis and prognosis [2]. KLK3 belongs to the kallikrein-related peptidase (*KLK*) multi-gene family which encodes fifteen homologous serine endopepti dases with trypsin or chymotrypsin-like substrate specificity. It is well documented that KLK proteases significantly contribute to several important (patho)physiological functions [3]. Consequently, there is a growing interest in the utility of KLKs in certain pathologies as biomarkers and therapeutic targets [4], [5], particularly in hormone-dependent cancers [6].

One KLK of interest, KLK4, is principally expressed in basal and secretory cells of the prostate gland and is commonly overexpressed in malignant prostate tumours [7], [8]. Recent studies indicate that the proteolytic activities of KLK4 closely align with events central to cancer development and progression. Firstly, KLK4 has been shown to degrade components of the extracellular matrix *in vitro* [9], as well as cleave insulin-like growth factor binding protein 3-6 [10] and urokinase plasminogen activator receptor [11]. Secondly, cell culture experiments have demonstrated that KLK4 enhances a diverse array of strongly tumourigenic functions. Of note, KLK4 stimulates protease-activated receptors −1 and −2 which are also overexpressed in prostate cancer, resulting in cytoskeletal remodelling and increased cell migration and proliferation [12], [13], [14]. These findings complement earlier studies which found overexpression of KLK4 was associated with an epithelial-to-mesenchymal transition in prostate cancer cells [8] and that KLK4 may modulate interactions between tumour cells and osteoblasts in the development of bone metastases [15]. Therefore, targeted inhibition of KLK4 may present an avenue to new treatments for advanced prostate cancer.

It has previously been reported that the naturally occurring sunflower trypsin inhibitor (SFTI-1) inhibits KLK4 [16], in addition to known targets such as trypsin [17], cathepsin G [18] and matriptase (ST14/MT-SP1) [19]. SFTI-1 is a 1.4 kDa cyclic Bowman-Birk serine protease inhibitor (BBI) isolated from sunflower (*Helianthus annuus*) seeds. Its three-dimensional structure in complex with trypsin [17] reveals a dual anti-parallel β-sheet arrangement stabilized by a disulfide bridge and an extensive internal hydrogen bonding network [20]. SFTI-1 binds to target proteases by an extended β-sheet across the P1-P4 residues to form a tight binding complex (trypsin/SFTI-1 *K*_i_ = 0.1 nM) [21]. This mode of binding is not only common to canonical serine protease inhibitors [22] but forms the basis of protein substrate and inhibitor recognition across all families of proteases [23].

Another important feature of SFTI-1 is that its scissile bond (P1–P1′) can be cleaved and reformed with an equilibrium of 1∶9 in favour of the intact bond [24]. This phenomenon is evident in at least 19 convergently evolved serine protease inhibitor families [25] and is referred to as ‘standard’ or Laskowski mechanism of inhibition [26], [27]. Standard mechanism binding loops are typified by a high degree of rigidity due to an internal network of stabilising hydrogen and disulfide bond(s). This has particular significance to inhibitor function; not only does it allow for a lower entropic debt upon protease binding [28], it also permits formation of an acyl-enzyme intermediate with largely unchanged conformation [29], [30]. As the products of hydrolysis are still associated with the protease, resynthesis of the peptide bond is more favoured than the intermolecular reaction with lower local reactant concentrations [30], [31].

To harness the favourable structural features of SFTI-1 and redirect inhibition towards KLK4, the contact β-sheet of SFTI was recently re-engineered using a sparse matrix peptide library to guide amino acid substitutions. The resulting inhibitor, SFTI-FCQR (P1 Lys to Arg, P2 Thr to Gln and P4 Arg to Phe) selectively inhibited KLK4 (*K*_i_ = 3.59±0.28 nM) and uniformly showed low inhibition of other SFTI-1 targets and closely related KLKs [16]. However, the constrained geometry of SFTI prevents the use of linear peptide libraries to optimise interactions beyond the P1–P4 residues, while producing a synthetic SFTI library is prohibitively costly and time consuming. Therefore, it is more practical to screen a virtual library of SFTI-FCQR variants.

Conventional *in silico* scoring functions rely on docking algorithms that treat the bonds of the ligand and receptor as rigid or semi-flexible to reduce the computational costs. However, these methods only have an acceptable degree of accuracy when considering ligands with few conformational states [32], [33]. Molecular dynamics (MD) offers a solution to the problem of structural flexibility. Indeed, several studies have successfully used MD to predict inhibitor performance, yielding new lead compounds or improving existing inhibitors [34], [35]. Recent advances in graphics card processors (GPUs) [36] have enabled GPU-implementation of MD algorithms, making them more accessible for flexible receptor-ligand analysis.

Here the GPU-implemented MD algorithms ACEMD [37] and NAMD [38] are used to explore the SFTI-1/trypsin complex and analyse an *in silico* library of SFTI-FCQR variants. Increased internal hydrogen bond frequency showed a high degree of accordance with enhanced inhibition *in vitro*. The most favourable substitution produced a second generation inhibitor with a binding affinity for KLK4 similar to that of SFTI-1 for trypsin while selectivity was markedly improved. These findings underline the importance of binding loop rigidity in canonical serine protease inhibitors and the need to maintain structural stability when modifying compounds of this class.

## Methods

### Protein expression and purification

Recombinant KLK4 and KLK14 were produced using Sf9 insect cell expression constructs as previously reported [14], [16]. These expression vectors generate the complete KLK amino acid sequence followed by a V5 epitope (GKPIPNPLLGLDST) and polyhistidine tags. Pro-KLKs were purified from conditioned media using Ni^2+^-nitrilotriacetic acid agarose (Qiagen) according to the manufacturer's instructions. After confirming the identity of purified proteins by Western blot analysis, pro-KLKs were aliquoted and stored at −80°C.

### Molecular dynamics

SFTI-FCQR variant/KLK4 complexes were generated by overlay of KLK4 (PDB ID 2BDG) and the trypsin/SFTI-1 complex (PDB ID 1SFI) in SPDBV v4.01 (RSMD 0.96 Å) [39] while mutations were made in YASARA Dynamics 9.12.13 [40]. Systems were solvated with TIP3P water and neutralized by Na^+^/Cl^−^ counterions to a final concentration of 100 mM in VMD 1.8.7 [41]. This generated systems of approximately 28000 atoms including 9000 water molecules.

Each protease–inhibitor complex was equilibrated using a stepwise relaxation procedure. In the first stage, all heavy-atoms were harmonically restrained with a force constant of 2 kcal/(mol Å^2^) before a conjugate gradient minimization of 5000 steps was applied using NAMD 2.6 [38] and CHARMM27 force fields parameters. This was followed by heating to 298 K before simulating 500 ps under NPT conditions with periodic boundary conditions. A Langevin thermostat with a damping coefficient of 0.5 ps^−1^ was used to maintain the system temperature. The system pressure was maintained at 1 atm using a Langevin piston barostat. The particle mesh Ewald algorithm was used to compute long-range electrostatic interactions at every time step and non-bonded interactions were truncated smoothly between 7.5 Å and 9 Å. All covalent hydrogen bonds were constrained by the SHAKE algorithm (or the SETTLE algorithm for water), permitting an integration time step of 2 fs. For the second stage, the restraints were retained on the protease and inhibitor α-carbons (Cα) only, while all constraints were released in the third stage.

Three independent production runs of 5 ns were carried out for each system using ACEMD [37]. These simulations were performed under NVT with otherwise identical force field and simulation parameters as above. Coordinates were saved every 500 simulation steps producing 5000 frames per run. Analyses were performed using VMD 1.8.7 with hydrogen bond lengths and angles set to 40° and 3.3 Å respectively, chosen to align with the reported trypsin/SFTI-1 complex [17].

### Synthesis of SFTI variants

All reagents were obtained from Auspep and all solvents from Merck unless stated otherwise. Inhibitors were synthesised as linear peptides on 2-chlorotrityl resin (1.3 mmol/g) derivatized with 0.9 mmol/g of the first residue, Ser (P1′). Coupling of the following nine residues was achieved using four-fold excess of Fmoc-protected amino acids dissolved in 0.25 M each of 2-(1H-benzotriazole-1-yl)-1,1,3,3-tetramethyluronium hexafluorophosphate (HBTU), 1-hydroxybenzo-triazole (HOBt), and N,N-diisopropylethylamine (DIPEA) in N,N-dimethylformamide (DMF). Fmoc protecting groups were removed by incubation in 50% piperidine and 5% 1,8-diazabicyclo[5.4.0]undec-7-ene (DBU) in DMF. Addition of the final four residues occurred as above, except that DMF was replaced with ‘magic mix’ solvent to prevent aggregation (Zhang et al., 1994). This contained equal parts of DMF, DCM and *N*-methyl-2-pyrrolidone (NMP) or DMF, toluene and NMP for coupling and Fmoc removal respectively.

Linear peptides were liberated from the solid support by successive changes of 0.5% trifluoroacetic acid (TFA) in dichloromethane (DCM). Cyclisation of the peptide backbone was achieved in solution using 125 mM each of 1-hydroxy-7-aza-benzotriazole (HOAt) and benzotriazol-1-yl-oxytripyrrolidinophosphonium hexafluorophosphate (PyBOP) dissolved in DMF containing 0.25 M DIPEA. Cyclization proceeded for 48 hr before dilution with an equal volume of DCM and extraction with H_2_O to remove residual reactants. Side chain protecting groups were removed from the dry product by cleavage for 2 hr in 93.75% TFA, with scavengers; 1.25% triisopropylsilane (TIS), 1.25% H_2_O and 3.75% thioanisole. Cleaved peptides were purified from remaining synthetic by-products by reverse phase HPLC (rp-HPLC) across a gradient of 20-100% isopropanol using a Jupiter 4 µ Proteo 90A C-18 column (Phenomenex). Formation of the internal disulphide bond was achieved by overnight stirring in an aqueous redox buffer (150 mM Tris-HCl pH 8.0, 1 mM EDTA, 10 mM reduced glutathione, 1 mM oxidised glutathione) while monitoring the reaction progress by MALDI-TOF mass spectrometry. Completed SFTI variants were purified, lyophilised and stored at -20°C.

### Synthesis of peptide substrates

Peptide *para*-nitroanilide (pNA) substrates were synthesised on *p*-phenylenediamine (Sigma-Aldrich) derivatised 2-chlorotrityl resin (1.3 mmol/g) according to previously described methods [16], [42]. Completed substrates were purified by rp-HPLC, validated by MALDI-TOF/MS and lyophilised before storage at −20°C.

### Inhibition assays

Bovine β-trypsin, bovine α-chymotrypsin and human thrombin were obtained from Sigma while KLK12 and matriptase were from R&D Systems. Increasing concentrations of inhibitors were incubated with various concentration of protease (final concentrations: KLK4, 1.5 nM; KLK12, 15 nM; KLK14, 2 nM; trypsin, 1 nM; matriptase, 4 nM; thrombin, 25 nM; α-chymotrypsin 25 nM) for 20 min in 200 µl assay buffer (100 mM Tris-HCl, 100 mM NaCl_2_, 0.005% triton-X, pH 8.0). Assays with thrombin and trypsin included 10 mM CaCl_2_. Enzyme activity was initiated by addition of substrate in 100 µl assay buffer (final concentration 100 µM; see Table 2). The rate of hydrolysis was measured at 405 nm over 7 min and was linear over this period. The extended assay period allowed for identification of inhibitors that were degraded. For SFTI-FCQR Asp_14_, *K*_i_ was determined by inhibition at various substrate concentrations using the competitive inhibition model and non-linear regression in Prism 5 (GraphPad Software Inc). The *K*_i_ for this inhibitor was also determined using the Morrison equation for tight binding inhibitors [43] (FVQR-pNA *K*_M_ = 679.9±113.1 µM [16]) and non-linear regression in Prism 5. Both methods produced comparable results (Table 2) and subsequent *K*_i_ values were determined with the Morrison method. Since SFTI-FCQR Asn_14_ had an IC_50_ below the concentration of KLK4, assays for this inhibitor were repeated with 0.15 nM KLK4 over 2 hr. The *k*_on_ and *k*_off_ were determined from the lag phases and steady state of inhibition as previously described [44] using assay conditions as above and 500 µM substrate. Assays for inhibition of fibrinogen proteolysis used the same buffer and enzyme concentrations as above with 7 µM fibrinogen substrate. Proteolysis proceeded for 15 min (trypsin), 90 min (KLK4 and 14) or 180 min (KLK12) before termination by boiling in SDS-PAGE sample buffer. Proteolysis fragments were separated on 10% polyacrylamide gels.

### Stability of SFTI variants in cell culture

The half-life in cell culture for SFTI-FCQR Asn_14_ and SFTI-FCQR Lys_14_ was determined using previously described methods [16]. Briefly, monolayers of LNCaP, 22Rv1 and PC3 cells were established in RPMI 1640 medium supplemented with 10% foetal calf serum (HyClone), 100 U/ml penicillin (Invitrogen) and 100 µg/ml streptomycin (Invitrogen). Cells were treated ±1 µM inhibitor (SFTI-FCQR Asn_14_ or SFTI-FCQR Lys_14_) in fresh serum-containing media. Samples of media were taken at 24 hr intervals and boiled at 97°C for 15 min to denature serum protein. Residual inhibition by SFTI-FCQR variants was determined in competitive kinetic assays (as above), adding a volume of media to give 10 nM SFTI-FCQR Asn_14_ or 25 nM SFTI-FCQR Lys_14_ at 0 hr. Media without inhibitor was used to adjust for any endogenous media inhibition and data represent the mean ± SEM of three triplicate experiments.

### Assessment of bioavailability in mice

Stability and bioavailability of SFTI-FCQR Asn_14_ *in vivo* was assessed in BALB/cFoxn1/Arc mice by oral, intravenous and intraperitoneal delivery (3 mg/kg). Inhibitor was dissolved in PBS at a concentration of 0.6 mg/ml prior to dosing. Serum levels of SFTI-FCQR Asn_14_ were subsequently measured by Liquid Chromatography-Mass Spectrometry (LC-MS) at Tetra Q laboratories (University of Queensland, Brisbane, Australia). This study was carried out in strict accordance to the recommendations of the Australian Code of Practice for the Care and Use of Animals for Scientific purposes (7th edition 2004) and the protocol was approved by the University of Queensland Animal Ethics Committee (ABS group) which assigned the project approval code TetraQ/479/09/Bluebox. All efforts were made to minimize suffering by experimental animals.

## Results

### Molecular dynamics reveals a reduction in internal hydrogen bonds for SFTI-FCQR Asp_14_ compared to SFTI-1

**Figure 1:**
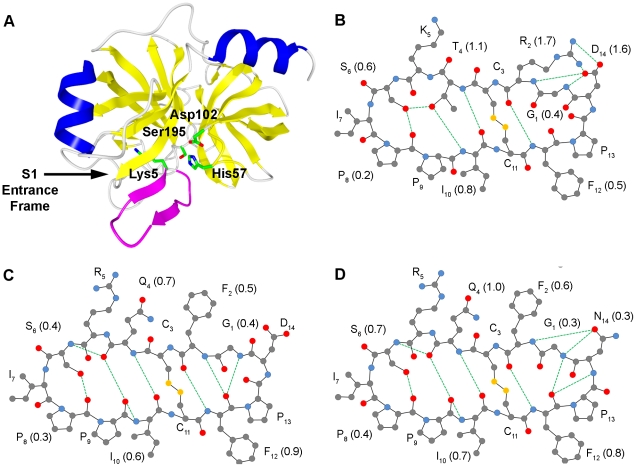
Representation of a trypsin/SFTI-1 complex and internal hydrogen bonding within SFTI variants during MD. Ribbon plot of SFTI-1 in complex with trypsin (A) with β-sheets and α-helices coloured in yellow and blue respectively, excluding SFTI-1 which is displayed in magenta. The residues of the catalytic triad of trypsin and the P1 Lys of SFTI-1 are shown in stick models with carbon in green, nitrogen in blue and oxygen in red. The structure of SFTI variants are shown in ball and stick 2D model with intramolecular hydrogen bond networks for (B) SFTI-1, (C) SFTI-FCQR Asp_14_ and (D) SFTI-FCQR Asn_14_. Amino acids are labelled with one letter code and residue number in subscript while the frequency of hydrogen bonds per residue is in brackets (rounded to nearest tenth). Carbons, oxygen, nitrogen and sulphur are represented by gray, red, blue and yellow respectively while hydrogens are excluded for clarity. Bond lengths and angles are intentionally unrealistic to enable easy viewing of hydrogen bonds, represented by dotted green line. Only hydrogen bonds occurring in more than 50% of trajectory frames are shown. Data is represented as mean from three independent 5 ns MD trajectories.

The contribution of various SFTI-1 residues to inhibitor rigidity and complex stability was examined by molecular dynamics simulations on the trypsin/SFTI-1 complex (Figure 1A; PDB ID 1SFI). Post-simulation analysis of the internal hydrogen bond network agreed with the reported structure [17] regarding the reactive loop while differing in the side loop (Figure 1B). Most notably, rather than acting solely as a proton acceptor for the backbone amide nitrogen atom of Arg_2_, the Asp_14_ side chain more often formed hydrogen bonds with the guanidino nitrogens of Arg_2_. These hydrogen bonds are evident in 20% of conformations in a solution structure of SFTI-1 [21]. Additionally, it appeared that the Asp_14_-Arg_2_ side chain hydrogen bonds subtly altered the backbone conformation meaning that the hydrogen bond between the amide of Gly_1_ and carbonyl oxygen of Phe_12_ seen in the crystal structure was infrequent.

**Figure 2:**
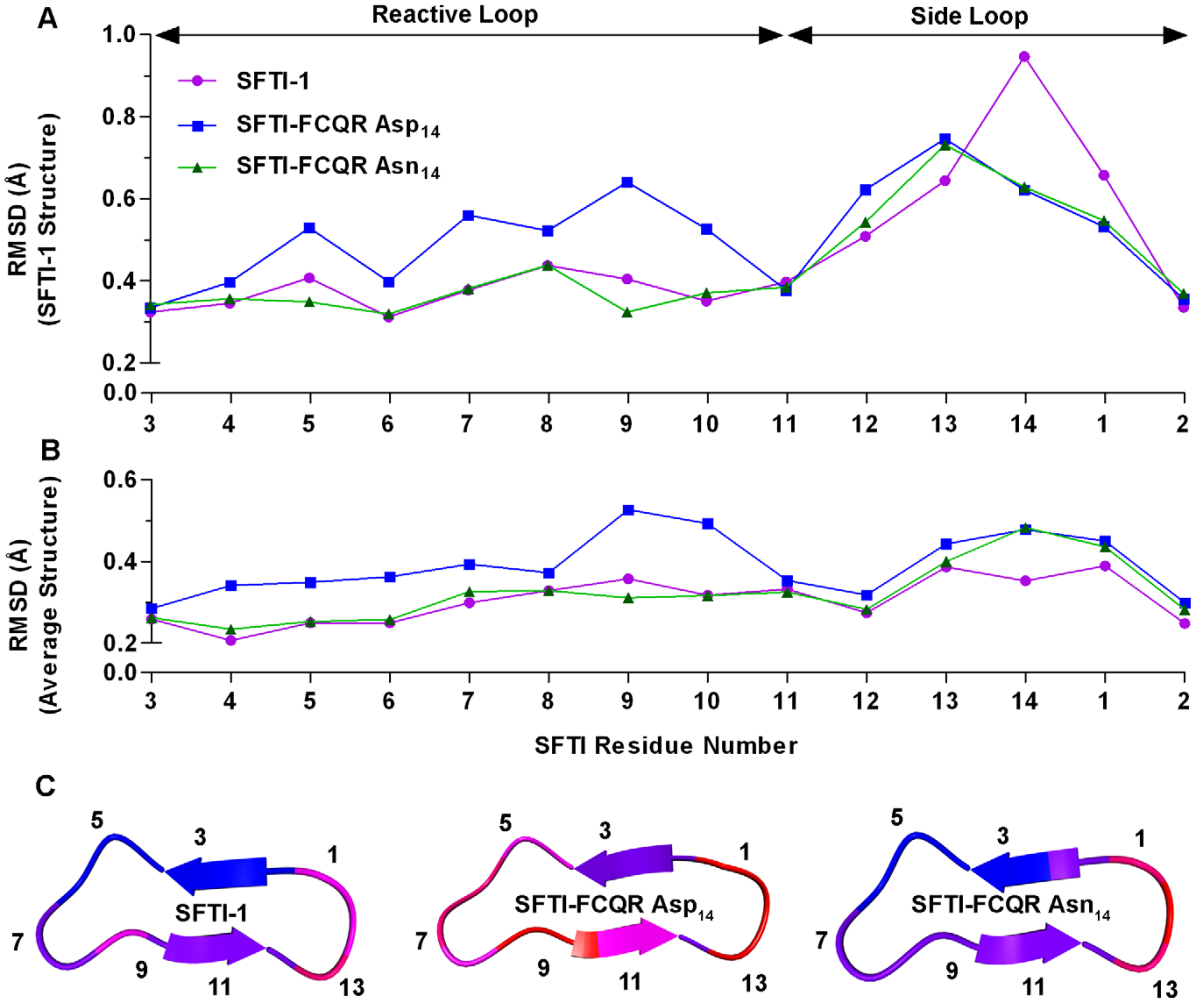
RMSD analysis for SFTI variants during MD. RMSD values between Cα of SFTI-1, SFTI-FCQR Asp_14_ and SFTI-FCQR Asn_14_ during MD and the (A) SFTI-1 starting structure or (B) calculated average simulation structures. (C) Ribbon plot showing the average simulation structures coloured according to Cα RMSD from low to high as blue, purple, magenta, orange, and red, labelled with odd residue numbers. Data is represented as mean from three independent 5 ns MD trajectories.

Consistent with this, the Cα RMSD values of SFTI-1 from the MD trajectory showed that the reactive loop conformation closely aligned with the starting structure while the side loop deviated markedly (Figure 2A). Determining Cα RMSD values using the simulation average structure as a reference indicated that although the change in conformation in the side loop varied from the SFTI-1 structure, the new conformation was stable (Figure 2B and C). The most rigid backbone atoms were found in residues that formed an extended β-sheet with trypsin (P3-P1) and as a result were flanked by both internal and intermolecular hydrogen bonds. Overall, the average number of internal hydrogen bonds for trypsin/SFTI-1 was 7.00±0.07, equivalent to the total number identified in the crystal structure.

Previously, SFTI-1 was re-engineered to produce a selective KLK4 inhibitor (SFTI-FCQR Asp_14_) by optimising protease/inhibitor interactions [16]. To examine the impact of modifying the contact β-sheet of SFTI on the distribution of internal hydrogen bonds, corresponding simulations were performed on KLK4 (PDB ID 2BDG) in complex with a model of SFTI-FCQR Asp_14_. The resulting analysis suggested a marked reduction (mean = 3.70±0.11) and rearrangement of the internal hydrogen bond network compared to SFTI-1 (Figure 1B). In the reactive loop, the side chain Thr_4_-Ser_6_ hydrogen bond was replaced by one between the carbonyl oxygen of Gln_4_ and the amide of Ser_6_ while Asp_14_ in the side loop no longer formed any recurrent internal hydrogen bonds. Perhaps as a result of the latter, the backbone hydrogen bonding pairs Phe_2_-Phe_12_ (Arg_2_-Phe_12_ in SFTI-1) and Gly_1_-Phe_12_ seen in the trypsin/SFTI-1 structure were again prevalent. These changes in internal hydrogen bonding pattern were accompanied by an altered conformation that poorly aligned with the SFTI-1 starting structure (Figure 2A) and reduced rigidity across the scaffold (Figure 2B and C).

### Substitution at Asp_14_ alters internal hydrogen bonding in SFTI

Inspection of the KLK4/SFTI-FCQR Asp_14_ simulation trajectories revealed that Asp_14_ showed a high level of disorder and was too far from KLK4 to make contact and thus appeared not to contribute to complex stability. This suggested that substitution of Asp_14_ could present an opportunity to restore the internal hydrogen bonding network of the inhibitor. Structural imperatives restricted the opportunities for further replacements around the SFTI backbone and so no further substitutional analyses of these positions were undertaken. Accordingly, a library of SFTI-FCQR variants containing all naturally occurring amino acids (excluding cysteine) at residue 14 was simulated followed by hydrogen bonds analysis (Table 1). The frequency of internal hydrogen bonds in the starting structure (SFTI-FCQR Asp_14_) was only slightly above the median. Further, modifying residue 14 had a considerable effect on the internal hydrogen bond network across the nineteen SFTI-FCQR variants, ranging from 2.28±0.07 (His_14_) to 4.29±0.31 (Asn_14_) average hydrogen bonds. In contrast, these substitutions had little effect on the number of intermolecular hydrogen bonds, producing a slight decrease for the majority of variants. To verify these *in silico* results, six variants representative of the diverse residue side chains and the number of hydrogen bonds were synthesised: Asn_14_ (amide), Tyr_14_ (aromatic), Lys_14_ (basic), Gly_14_ (flexible), Ala_14_ (less flexible) and Ser_14_ (alcohol). These were screened against KLK4 *in vitro* to determine respective inhibition constants (Table 2).

**Table 1:** *In silico* Internal Hydrogen Bond Analysis of SFTI-FCQR Residue 14 Variants.

SFTI variant	Internal Hydrogen Bonds (Mean ± SEM)	% Change from SFTI-FCQR Asp_14_	Intermolecular Hydrogen Bonds (Mean ± SEM)	% Change from SFTI-FCQR Asp_14_
**SFTI-FCQR Asn_14_**	4.68±0.086	26.5	8.46±0.10	2.1
**SFTI-FCQR Val_14_**	4.26±0.14	15.1	8.17±0.18	0.1
**SFTI-FCQR Tyr_14_**	4.07±0.24	9.9	8.22±0.30	−0.7
**SFTI-FCQR Met_14_**	3.98±0.12	7.6	8.11±0.20	−2.0
**SFTI-FCQR Lys_14_**	3.91±0.47	5.6	8.11±0.17	−2.1
**SFTI-FCQR Phe_14_**	3.89±0.15	5.1	7.58±0.08	−8.5
**SFTI-FCQR Ile_14_**	3.85±0.43	4.2	7.30±0.35	−11.8
**SFTI-FCQR Asp_14_**	3.70±0.11	0	8.28±0.19	0
**SFTI-FCQR Gly_14_**	3.67±0.30	−0.9	7.86±0.17	−5.1
**SFTI-FCQR Pro_14_**	3.66±0.43	−1.0	6.49±0.23	−21.7
**SFTI-FCQR Glu_14_**	3.59±0.50	−3.0	7.83±0.36	−5.5
**SFTI-FCQR Arg_14_**	3.51±0.51	−5.1	7.65±0.04	−7.6
**SFTI-FCQR Gln_14_**	3.36±0.25	−9.2	7.52±0.14	−9.2
**SFTI-FCQR Leu_14_**	3.33±0.26	−10.0	8.11±0.17	−7.7
**SFTI-FCQR Trp_14_**	3.12±0.22	−15.6	7.92±0.36	−4.3
**SFTI-FCQR Thr_14_**	2.93±0.093	−21.0	7.34±0.06	−11.3
**SFTI-FCQR Ala_14_**	2.89±0.085	−21.8	7.25±0.16	−12.4
**SFTI-FCQR Ser_14_**	2.56±0.048	−30.8	7.52±0.10	−9.1
**SFTI-FCQR His_14_**	2.28±0.068	−38.2	8.07 ± 0.11	−2.5

**Table 2:** Inhibitory Properties of SFTI-1, SFTI-FCQR and SFTI-FCQR Residue 14 Variants.

Enzyme	Inhibitor	IC_50_ (nM)	*K*_i_ (nM)	Morrison *K*_i_ (nM)	Theoretical Mass	Determined Mass	Substrate (100 µM)
**KLK4**	SFTI-FCQR Asn_14_	0.0635±0.0024	-	0.0386±0.0060	1559.75	1560.40	FVQRpNA
	SFTI-FCQR Tyr_14_	3.47± 0.20	-	2.55±0.43	1610.77	1610.56	
	SFTI-FCQR Lys_14_	6.07±0.13	-	3.56±0.27	1573.80	1574.93	
	SFTI-FCQR Asp_14_	7.97±1.08 [16]	3.62±0.26	3.89±0.40	1560.73	1559.94	
	SFTI-FCQR Gly_14_	14.74±1.089	-	10.39±2.87	1502.73	1504.77	
	SFTI-FCQR Ala_14_	26.23±0.85	-	18.31± 3.36	1516.74	1517.99	
	SFTI-FCQR Ser_14_	29.23±1.081	-	21.24 ±3.81	1532.74	1533.94	
	SFTI-1	221±10.1 [16]	-	-	1514.75	1514.84	
**KLK14**	SFTI-FCQR Asp_14_	1506±37.1 [16]	-	-	-	-	Ac-GSLR-pNA
	SFTI-FCQR Asn_14_	251± 21.9	-	-	-	-	
**β-Trypsin**	SFTI-FCQR Asp_14_	4064±109 [16]	-	-	-	-	BAPNA
	SFTI-FCQR Asn_14_	2178±145	-	-	-	-	
	SFTI-1	-	0.1 [17]	-	-	-	
**Matriptase**	SFTI-FCQR Asn_14_	>10,000	-	-	-	-	Bz-FVRpNA
	SFTI-1	-	0.92 [19]	-	-	-	N-t-Boc-QAR-AMC
**Thrombin**	SFTI-FCQR Asn_14_	>10,000	-	-	-	-	Bz-FVRpNA
	SFTI-1	-	5050 [19]	-	-	-	N-t-Boc-LRR-AMC
**Chymotrypsin**	SFTI-FCQR Asn_14_	>10,000	-	-	-	-	WpNA
	SFTI-1	-	2300±100 [51]	-	-	-	N-succinyl-AAPPpNA

Amino acids are represented by the one letter code.

**Figure 3:**
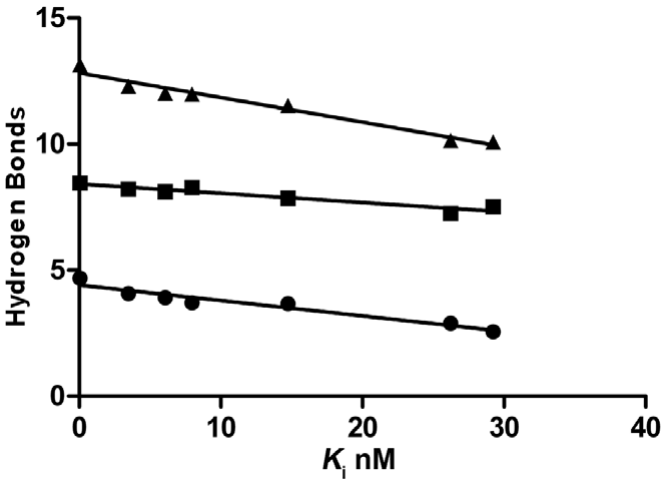
Relationship between *K*_i_ and number of internal hydrogen bonds. Plot of the average number of internal (circles), intermolecular (squares) and total (triangles) hydrogen bonds of SFTI-FCQR variants (Asn_14_, Tyr_14_, Lys_14_, Asp_14_, Gly_14_, Ala_14_ and Ser_14_) from Table 1 versus Morrison *K*_i_ values from Table 2.

SFTI-FCQR Asn_14_, predicted to have the most internal hydrogen bonds, was also the most potent KLK4 inhibitor with a *K*_i_ of 0.0386±0.0060 nM, exceeding that of SFTI-1 for trypsin (*K*_i_ = 0.1 nM). Furthermore, there was a consistent correlation between increasing number of internal hydrogen bonds during MD simulation and decreasing inhibition constants *in vitro* (Figure 3). However, it should be noted that SFTI-FCQR Lys_14_ assays were carried out immediately after addition of inhibitor since this variant was degraded after prolonged incubation with KLK4 (t½ = 56.3±6.2 minutes). This may reflect the introduction of a second potential cut site for trypsin-like proteases (Lys) on the side loop of SFTI. Although the Arg_5_-Ser_6_ scissile bond can be cleaved on SFTI without detrimental effect, the side loop does not have the features of a canonical loop and cleavage of the Gly_1_-Lys_14_ peptide bond may be irreversible.

**Figure 4:**
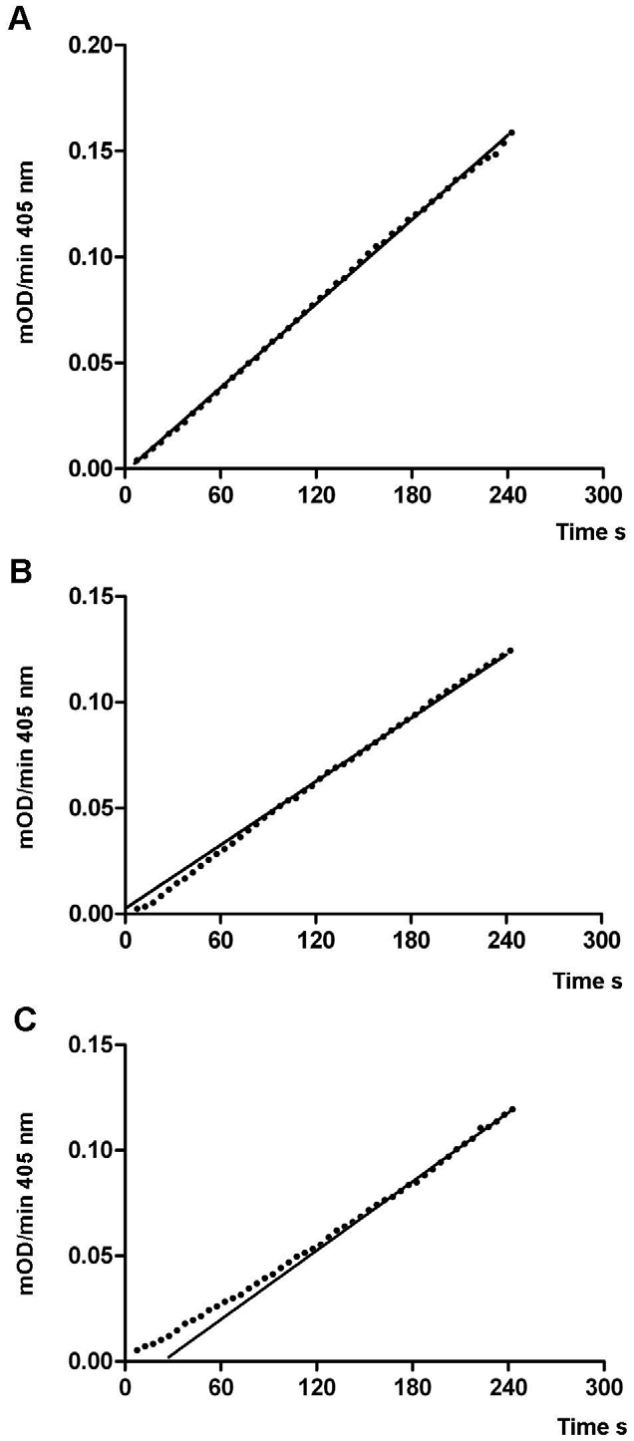
Assessment of *k*_off_ for SFTI-FCQR Asn_14_. Lag phases and steady state for inhibitor binding to KLK4: (A) uninhibited reaction progress (B) simultaneous addition of substrate and inhibitor (C) preformed enzyme inhibitor complex. The *k*_off_ rate was calculated graphically from the absolute difference between the steady states at y = zero. Rates shown are the average of three independent experiments.

Examining the internal hydrogen bonding network of SFTI-FCQR Asn_14_ revealed that substitutions at position 14 influenced their frequency and distribution across the entire scaffold (Figure 1C). In the side loop, the Asn_14_ side chain formed hydrogen bonds with the backbone amides of Phe_2_ and Gly_1_ with similar prevalence as seen for corresponding residues in SFTI-1. In comparison to SFTI-FCQR Asp_14_, the hydrogen bonds between Phe_2_-Phe_12_ and Gly_1_-Phe_12_ were similarly frequent while a further hydrogen bond was prevalent between the backbone amide of Asn_14_ and the carbonyl oxygen of Phe_12_. Overall, it appeared that changes in the hydrogen bonding pattern in the side loop of SFTI-FCQR Asn_14_ restored the frequency of the hydrogen bonds in the reactive loop to the level determined for SFTI-1. A previous study also reported that hydrogen bonds of the side loop, in particular the one formed between carboxylic oxygen of Asp_14_ and the Gly_2_ amide, were necessary to provide rigidity to the SFTI-1 reactive loop [20]. Consequently, this resulted in a reactive loop that closely aligned with the SFTI-1 starting structure both in terms of conformation (Figure 2A) and structural stability (Figure 2B and C). Consistent with a highly rigid scaffold, *k*_off_ was found to be 0.031±0.010 s^-1^ with a calculated second order rate constant (*k*_off_/*K*_i_) of 8.03×10^8^ M^−1^ s^−1^ (Figure 4), suggesting that SFTI-FCQR Asn_14_ binding to KLK4 is diffusion controlled. Collectively, these findings indicate that residue 14 of the side loop is instrumental for maintaining conformational stability of the SFTI reactive loop, a requirement for potent standard mechanism inhibition.

### SFTI-FCQR Asn_14_ is a selective KLK4 inhibitor

Screening SFTI-FCQR Asn_14_ against a panel of serine protease targets revealed that this variant was more selective than SFTI-FCQR Asp_14_. The most closely related enzyme to KLK4 is KLK14 with 85% sequence identity within 5 Å of the catalytic triad, while trypsin is a high affinity target for SFTI-1. Although SFTI-FCQR Asn_14_ more potently inhibited KLK14 and trypsin, the relative increase in inhibition was only six-fold and two-fold respectively, compared to 125-fold improvement for KLK4 (Table 2). This likely reflects that increasing hydrogen bonds, and therefore binding loop rigidity, produces a more potent inhibitor in general. Matriptase, thrombin and α-chymotrypsin which are also inhibited by SFTI-1, showed no inhibition with SFTI-FCQR Asn_14_ at 10,000 nM.

**Figure 5:**
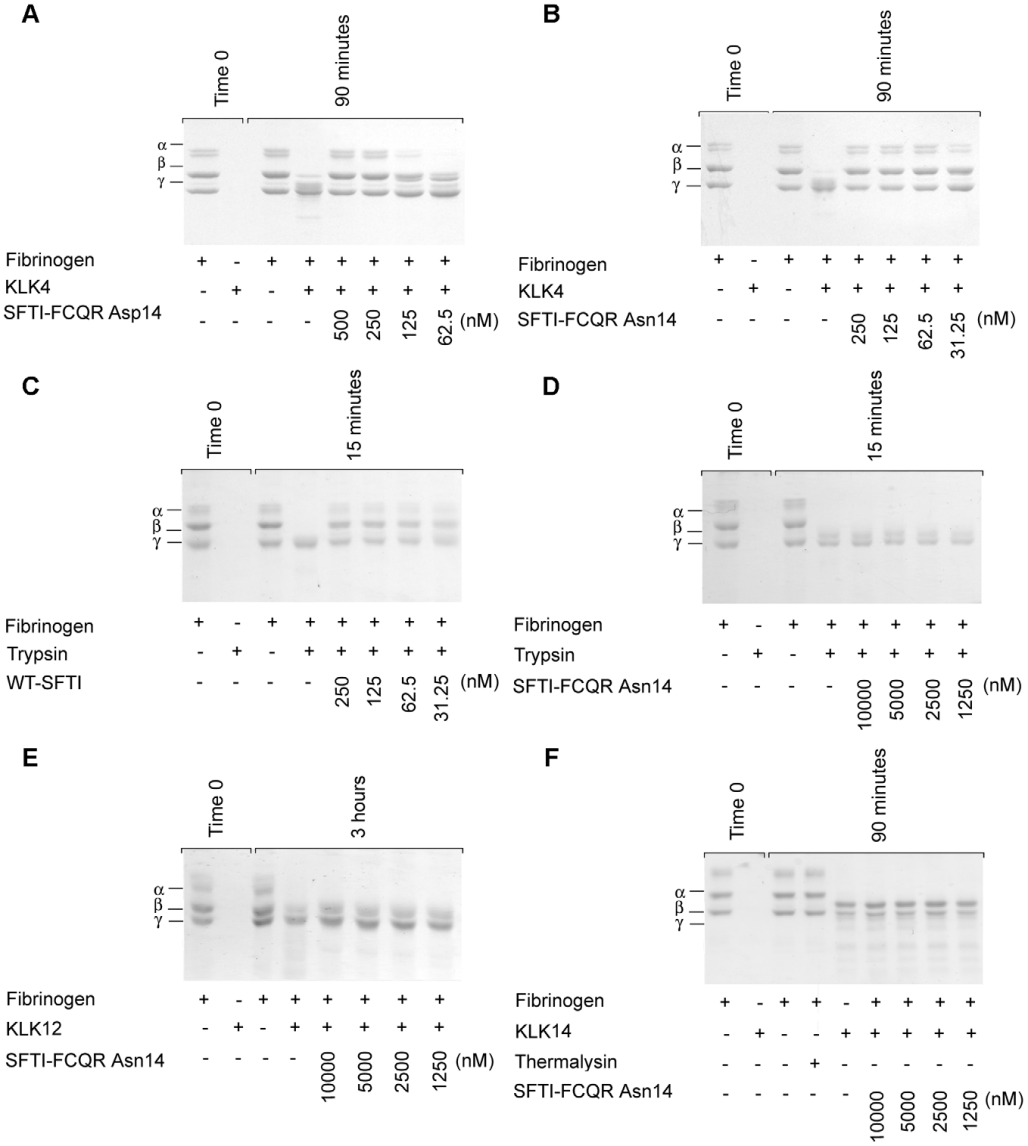
Selective inhibition of serine protease proteolytic activity by SFTI-FCQR Asn_14_. Examination of fibrinogen proteolysis by trypsin and kallikreins by SDS-PAGE. Bands were visualised with Coomassie blue staining after resolving on 10% polyacrylamide gels. Images are representative of three separate experiments. Inhibition of KLK4 proteolytic activity by (A) SFTI-FCQR Asp_14_ and (B) SFTI-FCQR Asn_14_. Inhibition of trypsin proteolytic activity by (C) SFTI-1 and (D) SFTI-FCQR Asn_14_. Inhibition of proteolytic activity of (F) KLK12 and (F) KLK14 by SFTI-FCQR Asn_14_.

Further, it has been demonstrated that amidolytic inhibition of a small peptide substrate does not necessarily equate to proteolytic inhibition. For example, SFTI-1 inhibits KLK4 in amidolytic assays (IC_50_ = 221±10.1 nM) but not in fibrinogen digestion assays with 2 µM inhibitor [16]. Consequently, the ability of SFTI-FCQR Asn_14_ to inhibit proteolysis of fibrinogen by KLK4, KLK12, KLK14 and trypsin was assessed. KLK4 proteolysis was blocked at inhibitor concentrations as low as 62.5 nM for SFTI-FCQR Asn_14_ compared to 250 nM for SFTI-FCQR Asp_14_, with more robust inhibition of degradation of the KLK4-preferred fibrinogen α-chain (Figure 5A–B). No inhibition of KLK12, KLK14 or trypsin fibrinogen proteolysis by SFTI-FCQR Asn_14_ occurred up to 10,000 nM (Figure 5C–F).

### SFTI-FCQR Asn_14_ is stable in culture with prostate cancer cells

**Figure 6:**
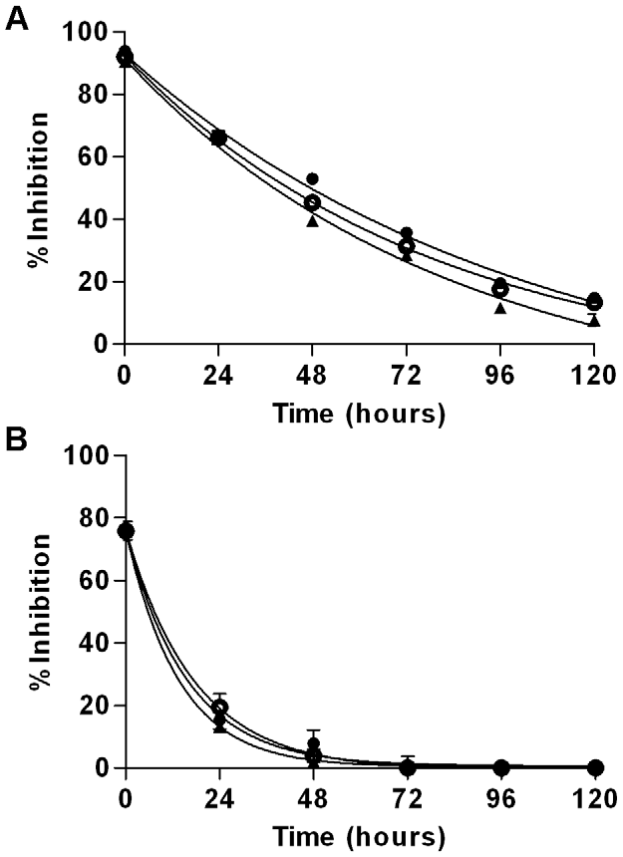
Stability of SFTI variants in contact with prostate cancer cells *in vitro.* Residual activity of (A) SFTI-FCQR Asn_14_ and (B) SFTI-FCQR Lys_14_ in cell culture media from prostate cancer cells treated with a single dose of inhibitor. Endogenous inhibitors were removed by boiling and centrifugation. Stability was assessed against LNCaP (closed circles), 22Rv1 (triangles), and PC3 cells (open circles). Data are mean ± SEM from three experiments in triplicate.

Previous evaluation of SFTI-FCQR Asp_14_ stability in culture with prostate cancer cells revealed that it was highly resistant to breakdown [16]. Whether replacing Asp_14_ with Asn markedly altered inhibitor stability in a cellular environment was assessed by calculating the half-life of SFTI-FCQR Asn_14_. Additionally, the SFTI-FCQR Lys_14_ half-life was determined given that this variant seemed to be degraded in competitive kinetic assays (see above). For SFTI-FCQR Asn_14_, inhibition of KLK4 gradually declined over time indicating a slow rate of decay that was comparable across each cell line (figure 6A). Despite an average reduction in stability compared to SFTI-FCQR Asp_14_, a half-life 55–70 hours is still well above the expected clearance time for peptide-based therapeutics *in vivo*. Further, in agreement with previous observations, SFTI-FCQR Lys_14_ was rapidly degraded with two-thirds of the initial activity lost within the first 24 hr (figure 6B).

### SFTI-FCQR Asn_14_ is bioavailable in mice when administered by intraperitoneal perfusion

**Figure 7:**
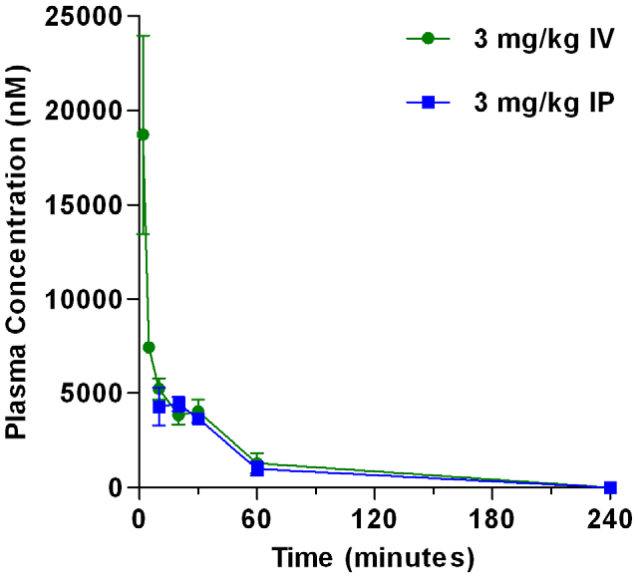
Bioavailability of SFTI-FCQR Asn_14_ in mice. Serum levels of SFTI-FCQR Asn_14_ administered at 3 mg/kg via the intravenous (IV), intraperitoneal (IP) routes in mice. Serum half life was 25-28 minutes with 10.0±0.8 nM inhibitor serum levels at 4 hours. The data is expressed as mean ± SEM (IV, n = 3; IP, n = 2).

While other BBIs are readily bioavailable [45] whether this applies to SFTI-1 or previously produced variants is yet to be determined. To establish the pharmacokinetic profile of SFTI-FCQR Asn_14_, the inhibitor was delivered via intravenous (IV), intraperitoneal (IP) and oral routes to BALB/c mice before serum samples taken over time were quantified by LC-MS. Orally delivered SFTI-FCQR Asn_14_ did not result in detectable levels of inhibitor in serum. In contrast IV and IP administered SFTI-FCQR Asn_14_ at a dosage of 3 mg/kg had a serum half-life of 25–28 mins with a residual inhibition concentration of 10.0±0.8 nM after 4 hours irrespective of delivery route (Figure 7).

## Discussion

This study has shown that when re-engineering a canonical serine protease inhibitor preserving binding loop rigidity and conformation is essential to maintain high binding affinity. Indeed, inhibitor variants with more frequent internal hydrogen bonds *in silico* correlated with more potent inhibition *in vitro,* emphasising their role in tight binding complexes. This guided production of an inhibitor with 125-fold improved potency for KLK4 and enhanced selectivity over off-target proteases, including closely related KLKs. Further, SFTI-FCQR Asn_14_ was stable in a cancer cell milieu and bioavailable by intraperitoneal perfusion in mice, making it an attractive candidate for further therapeutic development.

The suitability of SFTI as a generic scaffold for inhibitor design and its properties for maintaining structural integrity within a cell environment have previously been discussed in detail [4], [16]. Although SFTI-FCQR Asn_14_ was less stable than SFTI-FCQR Asp_14_ in culture with prostate cancer cells, it was sufficiently resistant to degradation, highlighting the robustness of the SFTI scaffold. The fact that SFTI-FCQR Asn_14_ was also bioavailable by IP indicates that the inhibitor had the ability to diffuse across tissues *in vivo*. This suggests that using a slow release depot implant is a viable mode of delivery. Alternatively there are numerous methods available for improving the retention of peptide drugs as previously reviewed [4]. Most notably, MD analysis indicated that Ile_10_ did not make contact with KLK4 and is positioned to provide an anchoring point for PEGylation [46] unlikely to markedly affect inhibitory properties.

Producing potent and selective standard mechanism inhibitors depends on fully realizing the highly conserved and successful structural features of the canonical loop. These inhibitors share two important properties. First, their reactive sites occur in constrained binding loops with similar structure and conformation [25]. Second, rigidity is maintained by intrinsic structural determinants allowing for positioning the P1′ free amine for peptide bond reformation after the scissile bond is cleaved [30], [31]. Structural comparison delineates 19 families of inhibitors (I1-I3, I7, I8, I10-13, I15-20, I36 and I40) that belong to 13 clans comprising distinct protein folds and evolutionary origins [25]. The fact that the conformation of the canonical binding loop has evolved numerous times independently highlights its versatility as a starting structure for inhibitor design. However, whilst most tight binding standard mechanism inhibitors with large, flexible contact surfaces are slow binding, the SFTI scaffold is reduced to a simple canonical loop allowing for both fast and tight binding.

Strategies to re-engineer serine protease inhibitors commonly focus on the active site binding β-sheet of the canonical loop. The previous production of a selective KLK4 inhibitor utilised this approach by grafting a preferred substrate sequence into the β-sheet of SFTI-1 [16]. While the importance of canonical loop rigidity is well appreciated in naturally occurring structures, it has not received similar attention when modifying these inhibitors for new targets. The present study focused on restoring the internal hydrogen bonding network of the SFTI scaffold, generating an inhibitor with considerably increased affinity. Indeed, modifications of residue 14 that enhanced internal hydrogen bonding increased potency of inhibition across all variants assayed *in vitro*. This may also explain the modest increase in affinity for trypsin and KLK14 by SFTI-FCQR Asn_14_. Consistent with the importance of residue 14 to hydrogen bonding, a previous study found that substituting Asp_14_ for Ala in SFTI-1 resulted in a marked reduction in potency for trypsin [47]. Thus two complementary strategies for enhancing inhibitor performance are evident; re-engineering the active site binding β-sheet of SFTI is instrumental in achieving selectivity while modulating the internal hydrogen bonding network engenders increased potency. Bringing these two components together in SFTI-FCQR Asn_14_ resulted in an inhibitor rivalling SFTI-1 in terms of potency without its promiscuity.

The findings presented here are in agreement with a previous *in silico* study on SFTI-1 using graph representation [48] to analyse how various internal hydrogen bonds contribute to structural rigidity [20]. Similarly, it was observed that the hydrogen bonds within the side loop were important for maintaining rigidity of the SFTI-1 reactive loop. The strongest contribution was conferred by the hydrogen bonding pairs of Asp_14_-Gly_1_ followed by Phe_12_-Gly_1_ and Phe_12_-Arg_2_. Although the findings from both studies closely align, graph representation is confined to structurally determined hydrogen bonding patterns. As a result, hydrogen bonds are either present or absent, preventing prediction of how subtle changes in these patterns and frequencies will affect binding affinities.

The prominent role of internal hydrogen bonding in maintaining canonical loop rigidity is not only limited to the SFTI scaffold. The same study by Costa and co-workers showed that hydrogen bonds are vital for stabilising the binding loop for BBIs (Family I12) in general [20]. Further, studies on Eglin C (Family I13) assessed point mutations by NMR and competitive binding assays, highlighting the relationship between inhibition constants and hydrogen bonds within the binding loop [49]. In fact the importance of the internal hydrogen bonding network in maintaining the conformation of the canonical loop has been demonstrated structurally across most families of standard mechanism inhibitors [50]. Preservation of these interactions is a key, yet often overlooked, property to consider when re-engineering this class of inhibitors.

In contrast to small-molecule inhibitors with few rotational bonds, conventional docking and scoring of protein-based inhibitors and their receptors have markedly lower rates of success [32]. The alternative is to calculate average binding affinities across MD trajectories including conformations of many low energy-state complexes [32]. These methods fail to recapitulate the most important aspect of standard mechanism serine protease inhibitors: the conformation and rigidity of the canonical loop before and after cleavage of the scissile bond as well as during the acyl-enzyme intermediate. Therefore, it is more effective to use computer-aided design methods to optimize structural features supporting the binding loop conformation to more closely replicate the native starting structure. This study focused on analysing one of these intrinsic canonical loop properties, namely hydrogen bonds, to accurately predict relative binding affinities of SFTI variants. We suggest that this simple approach is generally applicable as a tool to enhance binding affinity when re-engineering standard mechanism inhibitors. Used in conjunction with traditional peptide library screens to sample protease subsite preferences, this strategy is likely to produce both highly selective and potent inhibitors.
